# Beyond Smoking: Environmental Determinants of Asthma Prevalence in Western Nepal

**DOI:** 10.5696/2156-9614-10.25.200310

**Published:** 2020-02-28

**Authors:** Uttam Paudel, Krishna Prasad Pant

**Affiliations:** 1 Environmental Health Economist, Tribhuvan University, Nepal; 2 Visiting Faculty (Environmental Economics), Kathmandu University, Nepal

**Keywords:** Nepal, asthma, environment, household characteristics, probit model

## Abstract

**Background.:**

Asthma is widely prevalent in Nepal, but the causes are not well known aside from some general associations with ambient air pollution and microbial exposures. Information on the wide-ranging determinants of asthma prevalence among the population at risk can help policy makers to reduce risk.

**Objective.:**

The present study is a preliminary investigation of the environmental, socioeconomic and behavioral determinants of asthma prevalence in western Nepal.

**Methods.:**

A survey was conducted among 420 randomly selected households in western Nepal. A cross-sectional analytical study design was employed with the primary data using econometric tools of probit and logistic regression.

**Results.:**

Environmental variables such as extreme cold winter, deteriorating river water quality and air pollution were associated with an increase in asthma prevalence. However, individual or household characteristics such as advancing age of household head, use of pesticides in the home for the control of pests, piped drinking water with old pipes and lack of participation in awareness programs were associated with an increase in asthma prevalence.

**Discussion.:**

Among environmental factors, decreasing river water quality, increasing air pollution, and extremely cold winters are more likely to contribute to asthma prevalence. In light of the effects of environmental factors on the prevalence of asthma in Nepal, the high public and private costs of asthma could further impoverish the rural poor.

**Conclusions.:**

Environmental health policy makers should design adaptation strategies along with additional community programs addressing asthma-instigating factors. Programs to reduce environmental pollution can reduce morbidity due to asthma.

**Participant Consent.:**

Obtained

**Ethics Approval.:**

This study was approved by the Ethical Committee of the Nepal Health Research Council.

**Competing Interests.:**

The authors declare no competing financial interests.

## Introduction

Asthma is characterized by rescindable airflow obstacle giving rise to sporadic chest tightness, wheeze and breathlessness as a result of airway hyper-responsiveness.^1^ Asthma occurrence is affected by age and environment. Asthma is widespread, particularly among the older rural population in developing countries. Asthma in developed countries often begins in childhood or may develop in adulthood.^2^,^3^ In the global context, geographical differences in asthma prevalence are due to many powerful environmental impacts on its etiology.^4^ Asthma is primarily caused either by the effect of atopic allergen development or environmental exposures or both.^3^

A clinical review study found that the prevalence of allergic diseases due to exposures to microbial indoor allergens and house dust mites are the major cause of asthma development in childhood.^2^,^5^ Upon exposure to ambient air pollution, young children and those of lower socioeconomic status are highly susceptible to asthma. Those living in poverty have greater exposure to pollutants from traffic-related air pollution and environmental tobacco smoke in poor housing conditions.^5–7^ Other environmental factors such as outdoor air pollution, climate indicators, water pollution and human behavior have aggregate effects on asthma allergens, however, there have been few studies of these factors in developing countries.^8^,^9^

Asthma prevalence reports are now available from different sources around the world. As per the 2006 WHO report, asthma is responsible for 180,000 deaths annually and affects about 5% of the global population.^10^ In the United States, asthma prevalence increased from 7.3% to 8.4% of population from 2001–2011.^11^ Asthma prevalence reports in Africa and South Asia remain scarce, reflecting challenges in diagnosis of asthma due to weak access to health care facilities and asthma treatments in these regions.^12^ Family atopy and the presence of a cat at home are the major factors contributing to asthma prevalence in South Africa.^12^ In South American countries, the major environmental and socioeconomic determinants of asthma prevalence are water supply, sanitation, and the human development index and Gini index (income inequality).^13^ These studies, however, did not explore aggregate environmental and household determinants associated with asthma prevalence.

Despite the current progress in understanding the conditions and improvement in asthma in Asia, the major common challenges remain underexplored.^14^ The factors behind asthma heterogeneity and severe asthma exacerbation include progressive loss of environmental quality, uncontrolled air pollution, high prevalence of smoking, and poor asthma control programs. In South Asia, asthma prevalence in the general population is reported to be 6.3% in India, 10.7% in Bangladesh and 4.2% in Nepal.^6^,^15^ Dust allergy, food allergy and family history of asthma are common predictors of asthma prevalence in South Asia.^16^,^17^

In Nepal, there have been few studies exploring asthma prevalence due to indoor or outdoor air pollution.^18–20^ A recent study in Nepal found that the burning of dung briquettes for cooking had a significant effect on the prevalence of asthma and eye diseases with annual health costs of US $16.94 per household, which is 61% higher than the annual cost of using biogas (US $10.38), a cleaner alternative fuel for rural households.^21^ Previous studies have mainly focused on indoor and ambient air pollution as well as a few socioeconomic and health system variables in Nepal. As reported in previous international studies, asthma is also caused by environmental variables and other determinants, however, there have been few studies looking broadly at all possible factors potentially influencing the prevalence of asthma in Nepal or South Asia in general.^22^ Therefore, the present study aims to explore the environmental, socioeconomic and household behavioral determinants of asthma prevalence through a community-based survey in western Nepal.

**Abbreviations**
*CI*: Confidence interval
*GDP*: Gross domestic product
*LPG*: Liquefied petroleum gas
*SE*: Standard error
*VDC*: Village Development Committee
*WHO*: World Health Organization

## Methods

**Figure 1:**
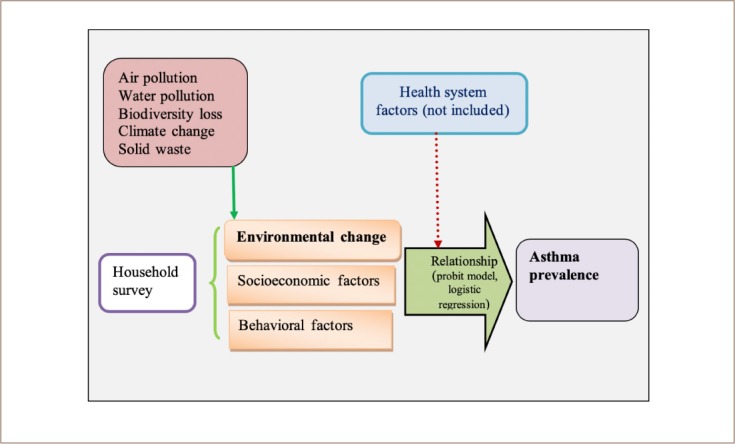
Conceptual framework

A cross-sectional analytical design was employed using primary data obtained from a household survey. After obtaining a sampling frame from a pilot survey, a simple random sampling method with systematic sampling with random start was employed to select a sample of 420 households. The questionnaire incorporates environmental, socioeconomic and behavioral variables, maintaining both qualitative and quantitative aspects *(Supplemental Material).* Data were collected through in-depth interviews of the head of sample households (head of household was defined as a senior member who manages financial affairs in the home). Household data were supplemented through focus group discussions with health professionals and community residents using a semi-structured questionnaire containing both qualitative and quantitative aspects. Information was also obtained from a review of documents from governmental and non-governmental sources. Collected data were compiled in the Statistical Program for the Social Sciences software program and transferred into the Stata software program for the analysis. Econometric theories were rigorously developed for the results of the logit model for the identification of the determinants of asthma prevalence in households. The design of the study is compressed in the conceptual framework presented in Figure 1.

### Study area

**Figure 2:**
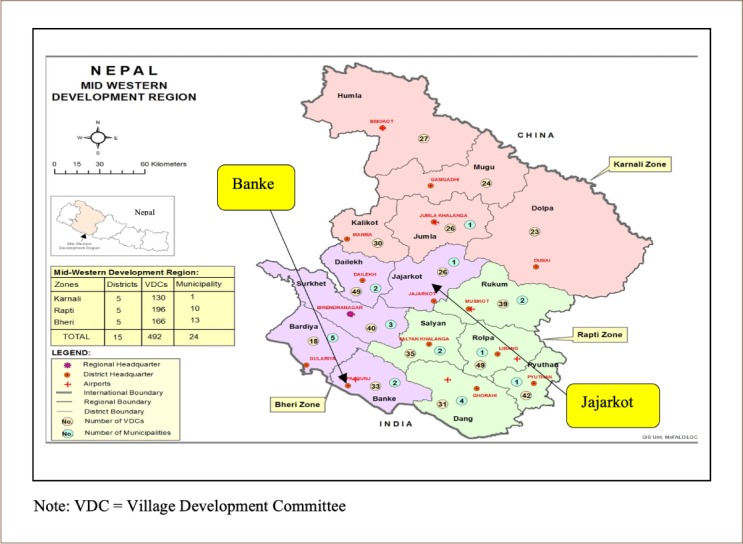
Map of study area (Source: MoFAGA, 2016)^24^

The study area was located in the Jajarkot and Banke districts *(Figure 2)* of western Nepal which join the higher mountains to the Terai region and have a high incidence and prevalence of disease. Western Nepal has the highest disease prevalence in the country.^23^ To address the disease situation at the country level, the two districts with the highest incidence of disease and that are highly sensitive to environmental changes were purposively chosen for the present study.

The Jajarkot district ranges in altitude from 610 m to 5440 m and is characterized by very poor socioeconomic status. The district has rugged mountains and transportation is very difficult due to limited bridges over rivers in the region. Increasing water shortages in high mountain villages and seasonal out-migration of labor, primarily to India, are also characteristic of the district. The Banke district, in the Terai region, is characterized by plains ranging from 109 m to 1950 m in altitude, with a climate ranging from subtropical to temperate. The socioeconomic status of the population in Banke is improved over that of Jajarkot. The population in Banke has better access to health and education services due to increased access to transportation and electricity. The high prevalence of disease in these districts and their surroundings is often reported by the news, but remains unaddressed in the scientific literature.

### Study setting and design

Prior to the final field survey, a rapid assessment study was done to pretest the questionnaire, ensuring good question flow, survey quality and to avoid any missing information. The Veri municipality in Jajarkot, the Nepalganj municipality, and the Janaki village municipality in the Banke district were selected for sampling. These municipalities were selected based on information provided by the district Public Health office based on their high incidence of disease and population heterogeneity. Next, a systematic sampling with random start was applied for selection of the sample households. The first household was selected randomly and five households were left between one sampling unit to another sampling unit (ie. every sixth dwelling was selected). The average distance between consecutive sampling units was roughly 500 m.

After the finalization of a semi-structured questionnaire *(Supplemental Material)*, a six-member team of public health graduates was selected as enumerators and trained on the study and questionnaire. The training covered the objectives of the study, questionnaire terminologies, and survey techniques. The aim of the study was to determine asthma prevalence due to changing environmental indicators over the last five years, with a focus on 2013 to 2017. A study protocol was developed and approval was granted by the Ethical Committee of the Nepal Health Research Council.

The data collection was performed through face to face in-person interview from 16 - 28 March 2018. Prior to the interview, verbal informed consent was obtained from each of the respondents after explanation of the objectives of the study and assurance that the study information would be used anonymously for research purposes only.

A field supervisor was deployed to ensure data quality and to minimize missing and incomplete information or irrelevant responses. To ensure the precision of the collected data, the team of enumerators went over the collected data. To supplement the data from the household survey, a key informant survey was performed with 20 community members aged 35 years or older and eight health professionals working in the district for triangulation of the data.

Previous studies have supported the assertion that indoor air pollution is associated with increasing asthma prevalence, however, asthma is affected by many other potential environmental variables.^3^,^9^ Based on the community survey, some of the relevant variables are hypothesized to affect asthma prevalence and are described with their hypothesized or expected sign (increasing (+ve) or decreasing (−ve) probability of asthma prevalence) in Table 1.

**Table 1:** Variables Hypothesized to Affect Asthma

**Variable**	**Expected sign**	**Mean**	**Standard deviation**
Winter temperature (decreasing =1, 0 otherwise)	+ve^8^,^25^	0.269	0.443
Outdoor air pollution (increasing=l, 0 otherwise)	+ve^9^,^18^	0.602	0.297
Heat wave (increasing= 1, 0 otherwise)	+ve^26^	0.821	0.269
River pollution (increase =1, 0 otherwise)	+ve^8^	0.704	0.396
Age of household head	+ve^27^	44.716	9.874
Drinking water source (pipeline water =1.0 otherwise)	−ve	0.527	0.432
Main cooking fuel (wood =1, 0 otherwise)	+ve^18^	0.786	0.329
Awareness program (not participated =1, 0 otherwise)	+ve^28^	0.392	0.290
Regular use of pesticides around home (yes = 1, no = 0)	+ve	0.354	0.479
Water purification devices at home (yes = 1, no = 0)	−ve^29^	0.109	0.312
Distance to the nearest market	+ve	5.085	6.212
Distance to the nearest health post	+ve	2.773	1.980
Water flow in pipe (decrease =1, 0 otherwise)	+ve	0.259	0.438

Abbreviations: +ve, increasing probability of asthma prevalence; −ve, decreasing probability of asthma prevalence

### Analysis

The present study explores the impact of environmental changes on asthma prevalence in western Nepal. Initially, to understand the impacts of environmental changes on the diseases, an unknown functional relationship was considered using Equation 1.

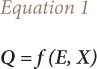
Where, Equation 1 links vectors of environmental variables (E) and socioeconomic and household behavioral variables (X) to asthma prevalence; Q. E includes selected environmental components, which are mutually exclusive. X includes household characteristics that may not be correlated with E and will not affect disease prevalence, possibly by conditioning environmental responses. A classic approach to estimating Equation 1 emphasizes spatial variation at a point in time. A linearized version of the above model can be found in Equation 2.

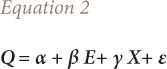
Where, Q is a categorical variable (asthma occurrence at household is coded as 1, and 0 otherwise). Therefore, a probit regression model was used to determine probable environmental and other factors attributable to asthma prevalence. The vector X typically includes several controls. Additional modeling information is given below.

Two models were run. Initially, independent environmental variables were chosen through an iterative process in model I. Subsequently, all environmental and other control independent variables were selected for model II. The correlation matrix was set for checking multicollinearity among the variables. Finally, all selected final variables were regressed with the binary disease prevalence variable to identify relevant environmental and other control variables.

### Econometric treatment

The probit analysis employed in this study is used to create a framework for probit analysis of health outcomes. Since the dependent variable is a binary random variable, a probit regression model is appropriate for the analysis of health outcomes. Following the model of McFadden, y_t_ is the binary random variable taking the value of 1 with probability P_t_, and follows Equation 3.^30^

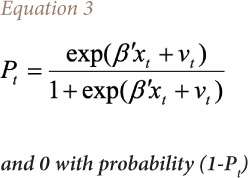
Where, *β* is the vector of unknown parameter, x_t_ is a vector of the known variables and v_t_ is a random variable with zero mean and constant variance *σ*^2^. If it is assumed that there are n_t_ independent observations on y_t_ denoted as y_t_(1), y_t_(2),……, y_t_(n_t_) Equation 4 is applied.

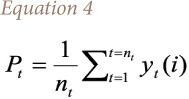
This model is different from the standard probit model in that v_t_ is included in the right-hand side of Equation 3. Following the Berkson approach used by recent study,^31^ then the probability function uses Equation 5.

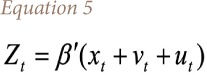
Where,

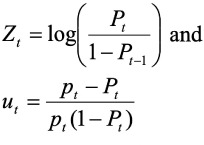
It is important to note that once the variable v_t_ in the above equation is assumed a random variable with zero mean and constant variance *σ*^2^, P_t_ applies to every observation *y_t_ (i), i =* 1,2*,....n_t_*. Therefore, sometimes P_t_ can be taken as an unknown parameter rather than random variables with zero mean and variance equal to 1/[*n_t_p_t_(*1*-P_t_)*], and *u_t_* and *v_t_* are uncorrelated because the conditional mean of *u_t_*, given *v_t_*, is zero. Thus, Equation 5 can be treated as a probit regression equation with heteroskedastic residuals, such that the variance of the i^th^ residual is equal to estimated value of coefficients.

In addition, the multiple logistic regression model is represented using Equation 6.

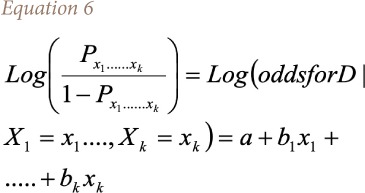
Here, *a* refers to the log odds of asthma prevalence *D* at the baseline level, indicating zero scales of all the risk variables. Moreover, the interpretation of the model is based on the log odds ratios in the form of value of the coefficients bi where i goes from 1 to n obtained from the one unit risk increase in the predictors x_1_, ……… x_k_ after the model run. For instance, b_1_ is the log odds ratio associated with a unit increase in the scale of X_1_, assuming all other risk variables in the model constant and no interaction exists.

Therefore, to identify the determinants of disease prevalence, the final multiple logistic regression equation for the present study uses Equation 7.

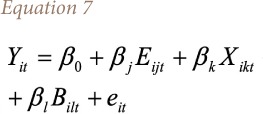
Where, Y_it_ is the household asthma prevalence at time interval t; is the constant coefficient; *β*_j_ is the vector of coefficients for environmental variables; *E_ijt_* is the vector of environmental variables at time t; *β*_k_ is the vector of coefficients of demographic and socioeconomic variables; *X_ikt_* is the vector of control (demographic and socioeconomic) variables; *β*_l_ is the vector of coefficients of household behavioral variables; *β*_ilt_ is the vector of household behavioral variables; and *e_it_* is a random error term.

This general model *(Equation 7)* is applicable for all of the particular probit regressions on particular asthma prevalence with all of the independent variables.

## Results

Of the total 420 respondents (heads of households), 56% were male and 44% were female. The respondents mainly belonged to three ethnicities: Brahmin/Chhetri, Dalit (formerly untouchables) and Janajati. Fewer Brahmin/Chhetri and Janajati females participated in the study compared to males. Other ethnicities include Muslims and some Terai castes.

The mean age of the head of household was 44.72 years (95% confidence interval (CI): 43.76 – 45.66, standard deviation = 9.87) ranging from 35 to 80 years. The average family size of the sample households was 6.5, which is quite a bit higher than the national average of 4.88.^32^ Family size in the southern part of western Nepal is higher than in the north. Regarding education of the head of household, the average education level was only 3.4 years of education *(Table 2)*, reflecting the low level of literacy for the population in this hilly poverty stricken region. Agriculture is the dominant occupation in the area, followed by services and businesses.

**Table 2:** Head of Household Demographics

	**Minimum**	**Maximum**	**Mean**	**Standard deviation**	**Skewness**	**Standard error**
Age (years)	35	80	44.72	9.88	1.04	0.11
Family size	1	35	6.55	3.49	3.04	0.18
Education (years)	0	14	3.39	4.31	0.88	0.12

Source: Field Survey, 2018

### Asthma-specific analysis

**Figure 3:**
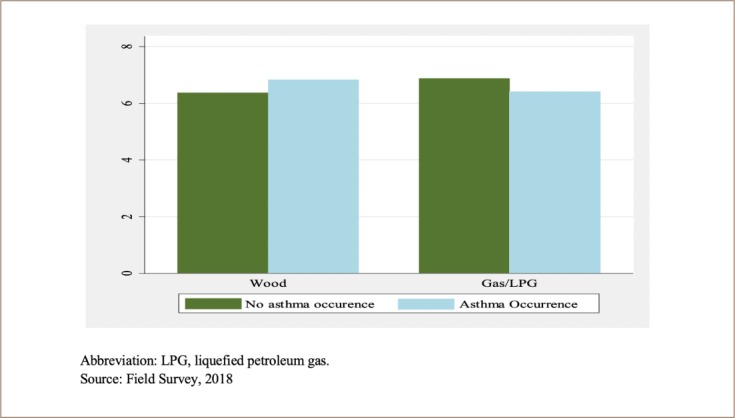
Distribution of asthma occurrence by main cooking fuel and average family size

Of the total respondents, 41% of households reported that at least one family member had experienced asthma. Of the total asthma patients who were regularly taking medicine, 4% were children, 74% are adult females and 22% were adult males. Almost 61% of cases came from hilly areas. About 78% of households whose main occupation was agriculture used wood as the main source of cooking fuel. Similarly, 78% of household heads were smokers. Only 17% of households had indoor smoke management systems with improved cooking stoves. Households with clean cooking stoves in the kitchen also used traditional stoves for preparation of semisolid feed for livestock (locally called *kundo*) from kitchen waste, maize flour, rice bran and other agricultural residues. As the quantity of *kundo* cooked is much larger than household food, firewood consumption is also quite large. There were relatively more asthma cases reported in households with a larger family size and greater production of *kundo* (produced by burning wood as fuel) which might be directly related to the number of milking buffaloes and cows *(Figure 3).*

Only 52% of households have access to piped water, and the rest use water from rivers and springs in the hills and tube-wells in the Terai. Nearly half of the respondents reported that they were aware of climate change. Similarly, 91% reported degradation of air quality and water quality, changes in temperature and rainfall patterns and loss of biodiversity over the last 15 years. Based on this community information, a detailed econometric analysis is presented below.

The correlation coefficients *(Table 3)* indicate an absence of multicollinearity problems among the independent variables, which calls for an econometric analysis method. The probit regression results in Table 4 indicate that lower winter temperature is likely to increase asthma prevalence in western Nepal compared to those unaffected by extreme cold temperatures, as supported by previous studies in the US and Denmark.^25^,^33^ The present findings show that with everything else held constant, a shift in winter temperature from normal to extreme cold conditions increased asthma prevalence by 22.7 (95% CI = 0.966–3.248).

**Table 3:** Correlation Matrix Among Hypothesized Variables

	**Ast**	**Wt**	**Ac**	**Ha**	**Wq**	**Pc**	**Wp**	**Ap**	**Age**	**Dw**	**Cf**	**Md**	**Dh**	**Pf**
**Ast**	1													
**Wt**	0.12	1												
**Ac**	0.03	−0.14	1											
**Ha**	0.07	0.07	0.05	1										
**Wq**	−0.07	−0.27	0.16	0.19	1									
**Pc**	0.01	−0.3	0.17	0.08	0.23	1								
**Wp**	−0.04	−0.09	0.06	−0.01	0.11	0.32	1							
**Ap**	0.07	0.04	0.04	−0.05	0.01	0.07	0.3	1						
**Age**	0.14	0.14	−0.06	0.01	−0.02	−0.02	0.05	−0.3	1					
**Dw**	0.12	0.23	−0.05	0.08	0.04	−0.31	−0.09	0.02	0.09	1				
**Cf**	0.05	0.13	−0.12	0.05	−0.09	−0.25	−0.33	−0.07	0.03	0.14	1			
**Md**	0.06	0.35	−0.08	0.08	−0.08	−0.31	−0.13	−0.05	0.06	0.25	0.17	1		
**Dh**	−0.02	0.18	−0.1	0.03	0.04	−0.1	−0.06	0.07	0.05	0.16	0.11	0.25	1	
**Pf**	0.07	−0.31	0.1	0.07	0.2	0	0.12	0.12	0.02	0.15	−0.02	−0.07	−0.05	1

Abbreviations: Ast, asthma; Wt, winter temperature; Ac, air conditioner; Ha, hot air; Wq, water quality in river; Pc, use of pesticides; Wp, water purification device; Ap, awareness program; Dw, source of drinking water; Cf, cooking fuel; Md, market distance; Pf, water flow in pipe; Dh, distance to hospital; Pf, pipe flow.

A positive value indicates a direct relationship among the variables and a negative value the opposite.

**Table 4:** Probit Regression

**Dependent variable: Asthma Occurrence (Yes=1, No=0)**			

**Variables**	**Coefficients model I (SE)**	**Coefficients model II (SE)**	**Odds ratio (95% CI)**
Decreasing winter temperature	0.298^**^ (0.151)	0.353^*^ (0.183)	1.772 (0.966–3.248)
Increasing air pollution	0.656 (0.264)	0.224 (0.240)	1.478 (0.657–3.323)
Heat wave	0.224 (0.134)	0.306 (0 .280)	1.752 (0.659–4.659)
Polluted river water	0.506 (0.274)	0.352^*^ (0.184)	1.565 (0.308–1.039)
Age		0.017^***^ (0.006)	1.030 (1.008–1.053)
Drinking water from pipes		0.297^*^ (0 .169)	1.659 (0.956–2.877)
Wood cooking fuel		0.140 (0.228)	1.241 (0.576–2.674)
No participation in awareness program		0.440^*^ (0.240)	2.105 (0.969–4.569)
Use of pesticides around home		0.414^**^ (0.171)	1.872 (1.129–3.513)
Water purification devices		−0.457^*^ (0.254)	0.465(0.198–1.093)
Distance to market		0.010 (0.012)	1.017 (0.979–1.056)
Distance to health center		−0.053 (0.037)	0.916 (0.810–1.035)
Pipe flow		0.344^**^ (0.169)	1.752 (1.004–3.057)

^***^ = p<0.01,

^**^ = p<0.05,

^*^ = p<0.1

Abbreviation: SE, standard error.

Unexpectedly, the estimated coefficient of air pollution was positive but insignificant in influence on asthma prevalence in western Nepal. Water pollution in rivers and rivulets around the community (which are the source of drinking water) is more likely to increase the probability of asthma in comparison to areas with better river water quality. Advancing age, no participation in an awareness program, use of pesticides around the home and drinking piped water (with rusted pipes) are more likely to increase the probability of asthma in western Nepal. For each unit increase in age, the odds of asthma prevalence was 1.27 (95% CI = 1.008–1.453). This confirms the prior expectation that increased age is associated with an increased chance of having asthma.

Awareness programs are also significant to reducing asthma in the study area. Those who participated in at least one health awareness program had a lower chance of developing asthma. Contrary to the theoretical expectation, piped drinking water was associated with an increase in asthma.

In addition, having at least one water purification device in the household decreases the probability of asthma by one half. As more than 80% of households use wood as the main cooking fuel, the effect of cooking fuel became insignificant on the asthma prevalence rate. Similarly, almost 80% of heads of households were smokers; therefore, smoking, which many studies have linked to asthma became insignificant and irrelevant to include in the list of affecting factors in the present study.^7^,^34^,^35^ All other things being equal, application of insecticides for control of insects and houseflies at home increased the odds of asthma prevalence by 1.87 (95% CI = 1.129–3.513) *(Table 4).*

## Discussion

The present study presents a detailed examination of the major environmental, socioeconomic and household behavioral determinants of asthma prevalence in western Nepal. Among environmental factors, decreasing river water quality and extremely cold winters are more likely to contribute to asthma prevalence. Use of pesticides to control insects at home was also linked with increased asthma. Piped drinking water supply was positively associated with asthma prevalence, consistent with the results of a Latin American study, while the availability of water purification devices was associated with a decrease in asthma cases.^13^ Advancing age and no participation in an awareness program were also linked with increasing asthma.

Decreasing winter temperature was strongly associated with an increase in asthma. This result is consistent with that of previous studies.^8^,^25^,^30^,^36^ Moreover, extreme cold weather in winter is associated with an increase in asthma.^37^ Increasing summer temperature also has the potential to change disease incidence patterns in Nepal, but this climatic factor seemed insignificant in the model to explain disease prevalence due to homogeneous responses of community people which did not create variations required for model fitting.

The use of river water for drinking purposes is common in some hilly areas of western Nepal. The link between asthma cases and deterioration of river water quality in western Nepal points to the need for measures to ensure the supply of safe drinking water. However, piped water was also positively associated with asthma prevalence. This might be due to contamination of water inside of old rusted iron pipes. Decreased water flow in pipes was more likely to be associated with increased asthma. This might be due to contaminated water inside pipes or inadequate amounts of water for family consumption. Extreme heat waves during the dry season did not significantly impact the incidence of asthma in this study; however, previous studies have shown significant effects.^38–40^ A European study reported results similar to those of the present study in the case of chronic respiratory diseases.^26^

A reduction in disease prevalence can be achieved through the use of water purification devices as water purification can clean up polluted water which may contain bacteria, nitrates and other disease-spreading agents. Decreasing water contamination can decrease the cost of water-borne diseases, as well as asthma.^29^ Awareness programs in these rural areas were also found to reduce disease prevalence, consistent with previous studies that found that awareness programs were associated with a decrease in asthma.^28^,^41^,^42^ Unexpectedly, this study shows that cooking fuels are insignificant to influence the asthma prevalence; however, a study in Nepal suggested that using wood for cooking fuel reduced asthma due to the heat generated from the burning of firewood.^19^

Increased age was associated with an increased chance of asthma, as found in previous studies.^27^,^43–46^ One prominent finding in Asian adults is that asthma prevalence increases with advancing age.^14^ This might be due to increased exposures to smoke inside the home and lowered access to healthcare during cold seasons due to the distance to health care facilities and household poverty. On the contrary, a study based on childhood asthma in the UK claimed that the risk of asthma prevalence lowered with age.^1^ Use of pesticides in the control of insects and pests around the home is reported to increase asthma risk.^47^,^48^ This confirms the theoretical expectation that direct and indirect exposures to insecticides would exacerbate asthma.

Asthma prevalence is directly associated with private and public cost burdens, estimated to be 149% of GDP per capita per healthy life years for mild asthma estimated by the WHO in Kenya, another developing country with similar healthcare access issues.^49^ Another study projected that the cost of asthma could increase by more than 5% due to climate change.^50^ A study based in Nepal estimated that the asthma treatment cost per case is US $73.78 without including the costs of lost productivity.^51^ The per case cost of asthma is quite high in developed countries, estimated to be US $420 in 2006 in the US.^52^ As environmental factors impact the prevalence of asthma in Nepal, investments in environmental improvement programs along with community awareness programs may improve the incidence of asthma and other diseases.

Finally, this study is consistent with previous studies and has comprehensively explored a potential link of environmental factors with the prevalence of asthma in Nepal, leading to increasing health costs associated with natural hazards. With regard to policy issues, the interconnectedness of asthma with the health of the natural environment is an relationship formally acknowledged by the WHO.^23^ This is the time to take immediate action on protection of the environment, as the health of the ecosystem and natural resources such as soil and water is linked to human health. Based on the results of this research, the government of Nepal along with other global cohorts should implement rural asthma case reduction programs through protection of the environment, which has a direct effect on chronic human respiratory diseases.

### Limitations

There are some limitations to the present study. This study did not include some environmental components such as improper land use, passive smoking and extreme weather events (apart from temperature) which might have potential effects on human health and did not fully explore behavioral risk factors such as active smoking. This study is based on the response of community residents. It did not collect any experimental evidence such as indoor air pollution monitoring. The study covered only two districts in western Nepal in a cross-sectional study design, and therefore the results cannot be generalized to the entire country of Nepal. Air quality data were not included due to the unavailability of air monitoring stations in the study area.

## Conclusions

The present study is a preliminary assessment of environmental change and its effects on asthma prevalence using the econometric probit regression method of analysis. Analysis of the determinants of disease prevalence found that environmental variables such as extremely cold winter weather, polluted air and polluted water in the river were associated with the increase of asthma prevalence in western Nepal. Measures are needed to provide cleaner heating systems for households, masks or other means for breathing when exposed to polluted ambient air and treatments to clean river water in order to reduce the prevalence of asthma in this region. Similarly, renovation of drinking water pipes or a clean piped water supply, availability of water purification devices, and adequate awareness programs are promising factors and practices to reduce asthma prevalence. An integrated environment adaptation program together with measures addressing household-level socioeconomic and behavioral factors could significantly reduce the prevalence of asthma. Policymakers should adopt an adaptation strategy addressing these environmental factors in order to mitigate the human health costs of asthma.
