# Supplementary material for: Beyond Smoking: Environmental Determinants of Asthma Prevalence in Western Nepal
Source: J Health Pollut. 2020 Feb 28;10(25):200310. doi: 10.5696/2156-9614-10.25.200310 (PMC7058133; doi:10.5696/2156-9614-10.25.200310)
Supplement: Supplementary file 1 [file Paudel_Supplemental_Material.doc]

**Supplemental Material**

**Household Survey Questionnaire**

The objective of this survey is to identify factors (environmental, social, economic and behavioral) that affect disease prevalence in midwestern Nepal and to estimate the societal cost burden in affected areas. To fulfill this objective, Tribhuvan University is asking all stakeholders to provide information in this survey**.** One person from each household who is over 35 years of age and who has resided in the community for the past 15 years is requested to answer the following questions:

**Module 1: Introductory Information**

| **Introduction** | | | | | | **Information of the respondent** | | | | **Sex: Male-1, female-2** | **Age (years)** |
| --- | --- | --- | --- | --- | --- | --- | --- | --- | --- | --- | --- |
| **Identifying the sampling area** | | | | | | **A06**. Name of household head | | ……………… | |  |  |
| **A01**. District- | | …………… | | | | **A07**. Name of respondent (>35 years | | ……………… | |  |  |
| **A02**.VillageMU/Municipality | |  |  |  |  | **A08**. Contact number of respondent | | ………………………………… | | | |
|  |  | ……………. | | | |  |  |  |  |  |  |
| **A03**. Ward no: | | ………….. | | | | **A09**. Ethnicity of respondent | | Code: | (Advantaged-1, Janajati-2, Dalit-3, Others-4 (Specify……………..) | | |
|  |  |  |  |  |  | **A10**. Household size | |  | | | |
|  |  |  |  |  |  |  |  |  |  |  |  |
| **A04**. Name of village/tole | | ………….. | | | | **A11**. Education level of respondent | | Code: | (Class: 1, 2, 3, ….., …. 12, Bachelor, Higher, 0=Illiterate) | | |
| **A05**. Altitude/elevation of study area | |  |  |  |  | **A12**. Duration of stay in this community (in years) | |  | | | |
|  | |  | |  | | **A13**. Marital status of respondent | |  | | | |
|  | **Name:** | | | | |  |  | Code: | Married-1, Unmarried-2, Widow-3, Divorced-4, Separated-5 | | |
| **Enumerator** | **Signature:** | | | | | **A14**. Main current occupation | | Code: | Agriculture -1, Service -2, Business -3, Student-4, Searching job-5, others-6 | | |
|  | **Date:** | | | | |  |  |  |  |  |  |
| **Data entry operator**  **Name: Signature:** | | | | | | **Supervisor** | **Name:**  **Signature:** |  | | | |

**Module 2: Household Information**

| **Q.No.** | **Description** | | **Code** | **Q.No.** | **Description** | | **Code** | |
| --- | --- | --- | --- | --- | --- | --- | --- | --- |
|  |  | |  |  |  | |  |  |
| **B01** | Ownership of residence? | |  | **B02** | Type of infrastructure of your main residence. | |  |  |
|  |  |  |  |  |  |  |  |  |
|  | 1. | Owned |  |  | 1. | Permanent |  |  |
|  | 2. | Rented |  |  | 2. | Semi (half)-permanent |  |  |
|  |  |  |  |  |  |  |  |  |
|  | 3. | Institutional |  |  | 3. | Kachchi |  |  |
|  | 4. | Others (Specify)……………… |  |  |  |  |  |  |
|  |  |  |  |  | 4. | Others (Specify)…………………………. |  |  |
|  |  |  |  |  |  |  |  |  |
|  |  | |  |  |  | |  |  |
| **B03** | Sources of drinking water (rank three main | |  | **B04** | Sources of fuel used for cooking | |  |  |
|  |  |  |  |  |  |  |  |  |
|  | sources by priority) | |  |  | (three main sources by priority) | |  |  |
|  | 1. | Pipeline |  |  | 1. | Firewood |  |  |
|  |  |  |  |  |  |  |  |  |
|  | 2. | Shallow/Deep tube well |  |  | 2. | Gas/LPG |  |  |
|  |  |  |  |  |  |  |  |  |
|  |  |  |  |  |  |  |  |  |
|  | 3. | Well/ spring |  |  | 3. | Cow dung |  |  |
|  |  |  |  |  |  |  |  |  |
|  | 4. | Canal/River/stream water |  |  | 4. | Biogas |  |  |
|  |  |  |  |  |  |  |  |  |
|  | 5. | Rainwater |  |  | 5. | Kerosene |  |  |
|  | 6. | Spring/stone spout |  |  | 6. | Electricity |  |  |
|  |  |  |  |  |  |  |  |  |
|  | 7. | Jar/tanker |  |  | 7. | Briquette/rice husk |  |  |
|  | 8. | Others (Specify)………………… |  |  | 8. | Others (Specify)……………………………. |  |  |
|  |  | |  |  |  | |  |  |
| **B05** | Main source of energy for light | |  | **B06** | Main type of toilet/latrine facility | |  |  |
|  |  |  |  |  |  |  |  |  |
|  | 1. | Electricity |  |  | 1. | Flush toilet (connected to sewage) |  |  |
|  | 2. | Solar Power |  |  | 2. | Flush toilet (connected to septic tank) |  |  |
|  |  |  |  |  |  |  |  |  |
|  | 3. | Kerosene |  |  | 3. | General household latrine |  |  |
|  | 4. | Bio-gas |  |  | 4. | Public toilet |  |  |
|  | 5. | Others (Specify)…………………. |  |  | 5. | Open space/No latrine |  |  |
|  |  | |  |  |  | |  |  |
| **B07** | Sources of income of household in the | |  | **B08** | Which of the facilities listed below are family using: | |  |  |
|  |  |  |  |  |  |  |  |  |
|  | past 12 months (rank three main sources by priority) | |  |  | (multiple answers are possible for this question) | |  |  |

| **B11** | Do you have your own land? (Yes-1, No-2) |  | **B12** | If yes, area of land …………………Ropanies |  |
| --- | --- | --- | --- | --- | --- |
| **B13** | Is your land rented out? (yes-1, No-2) |  | **B14** | If yes, area of rented land …………..Ropanies |  |
| **B15** | How much did you receive from rented land last year? | …… | **B16** | Did you receive money from rented out tractors/crafted animals last year? (Yes-1, No-2), If yes, list amount | …….. |

**Module 3: Household Income Information (Over the past 12 months)**

| **Agricultural products** | **Net Income (Rs.) C01** | | **Livestock** | **Net Income (Rs.) C02** | | **Agriculture and forest** | **Net Income (Rs.) C03** | | **Others and business** | **Net Income (Rs.) C04** | |
| --- | --- | --- | --- | --- | --- | --- | --- | --- | --- | --- | --- |
|  | Income | Exp. |  | Income | Exp. |  | Income | Exp. |  | Income | Exp. |
| Rice |  |  | Goat/Sheep |  |  | Wood |  |  | Salary |  |  |
| Maize |  |  | Buffalo |  |  | Straw |  |  | Wage |  |  |
| Millet |  |  | Cow |  |  | Firewood |  |  | Business/Enterprises |  |  |
| Wheat |  |  | Ox |  |  | Dung cake |  |  | Bank interest |  |  |
| Soyabean |  |  | Chicken |  |  | Fruit tree wood |  |  | Loan interest |  |  |
| Pulse |  |  | Peason |  |  | Animal dung |  |  | Commissions and royalties |  |  |
| Potato |  |  | Duck |  |  | Wood log |  |  | Shares profit |  |  |
| Jute |  |  | Changra |  |  | Rented land |  |  | Penson |  |  |
| Faper |  |  | Pig |  |  | Dairy products |  |  | Social security allowances |  |  |
| Vegetables |  |  | Mule |  |  | Milk |  |  | Government in-kind transfer |  |  |
| Mustard/Jau |  |  | Rabbit |  |  | Coffee/Sugarcane |  |  | Gifts from other households |  |  |
| Fruits |  |  | Fish |  |  | Cardmom/Yarsa |  |  | Sales of property |  |  |
| Others |  |  | Honey |  |  | Others |  |  | Others |  |  |
| Total: |  |  |  |  |  |  |  |  |  |  |  |

| **C05.** | Distance to nearest market from your house | ....... km |  |
| --- | --- | --- | --- |
| **C06.** | Distance to nearest agriculture support center or livestock service center from your house | ....... km |  |
| **C07.** | Current farm mechanization | Yes = 1, No=2 |  |
| **C08.** | Farming experience of household head (years) | ……….Years |  |
| **C09.** | Smoking habit of household head/member inside home | Yes = 1, No=2 |  |

**Module 4: Household Access to Socioeconomic Services**

|  | Description | Response | Code |
| --- | --- | --- | --- |
| **D01.** | You or any family member is a member of saving & cooperative or micro financial institutions or in informal saving groups | Yes=1 |  |
| **D02.** | You or any family member has regular saving in the saving & cooperative or micro financial institutions or in informal saving groups | Yes=1 |  |
|  | over the past 12-month period | No=2 |  |
|  |  |  |  |
| **D03.** | You or any family member is a member of any other community-based organization (CFUG, MGs, etc.) | Yes=1 |  |
|  |  | No=2 |  |
| **D04.** | Your household received services from agriculture support center(s) and livestock service center over the past 12-month period | Yes=1 |  |
|  |  | No=2 |  |
|  |  |  |  |
| **D05.** | Distance to motorable road from your house | ….... km |  |
|  |  |  |  |
| **D06.** | Distance to nearest health center from your house | ……..km |  |
|  |  |  |  |
| **D07.** | Distance to nearest secondary school from your house | ……..km |  |
|  |  |  |  |

**Module 5: Basic Information about Temperature and Rainfall**

| **Temperature/**  **Rainfall** | **Temperature and rainfall compared to previous 15 years (Increased=1, Decreased-2, Remained same=3)** | | |  |  | **E05. Duration** | |  |  |
| --- | --- | --- | --- | --- | --- | --- | --- | --- | --- |
|  |  |  |  | Previous 15 years | |  |  | Now |  |
| **E01**. Summer temperature | …………........ | |  | Start date | Month | Week | Start date | Month | Week |
|  |  |  |  |  |  |  |
|  | ………………. | | |
| **E02**. Winter temperature |  |  |  |  |  |  |
|  |  |  |  |  |  |
|  |  |  |  |  |  |
|  |  |  |  |  |  |
| **E03**. Monsoon rainfall | ………………… |  |  |  |  |  |  |  |  |
|  |  |  |  |
|  | ………………. |  |  |  |  |  |  |  |
| **E04**. Winter rainfall |  |  |  |  |  |  |
|  |  |  |  |  |  |  |  |  |

**Module 6: Knowledge and Perceptions of Climate Change and Causes**

| **Description** |  | **Code** | | **Description** |  | **Code** |
| --- | --- | --- | --- | --- | --- | --- |
|  |  |  |  |  | |  |
| **E06.** Have you heard about climate change? |  |  |  | **E07.** If yes, what is your main source of information about climate change? | |  |
|  |  |  |  |
| *Yes…….1* |  |  |  | Radio….1 | Television……...2, |  |
| *No…….2* |  |  |  | Newspaper/publications….3 | Awareness campaign…4 |  |
|  |  |  |  |
| *If no, go to question F01* |  |  |  | Local bodies/authorities…5 | Neighbor and friends…6 |  |
|  |  |  |  | Family member….7 | Others….8 |  |
| **E08.** Do you think the climate of this area is different |  |  |  | **E09**. What is the main reason for climate change? | |  |
|  |  |  |  |
| than it was 15 years ago? |  |  |  | Deforestation…1 | Natural reasons…2 |  |
| *Yes….1,* |  |  |  | Industrialization…3 | Urbanization…4 |  |
|  |  |  |  |
| *No….2,If no, go to module 6.1* |  |  |  | Overuse or misuse of resources 5 | God’s wish…6 |  |
|  |  |  |  | Do not know…7 Others….8 | ***(Please select 3 options based on priority)*** | |

**Module 6.1: Information on Impact of Climate Change**

| S.N. | Natural disasters or events  due to climate change  (first identify the main  disaster events of the  locality) | Have you experienced any changes in the following events in this area over the past 15 years? | If yes, how has it  changed over the past  15 years | To what extent have these events  affected you or your family over the past 15 years? | What are the main reasons  for the occurrence of the following events over the past 15 years? |
| --- | --- | --- | --- | --- | --- |
|  |  | Yes…1, No…2, next column  Not applicable…3, next column | Increased …. 1  Decreased … 2 | Very low …1, Low … 2, Moderate … 3, High ….4,  Very high … 5 | (write the codes of the main three  reasons by priority)* |
|  |  | **F01** | **F02** | **F03** | **F04** |
| 1 | Drought |  |  |  |  |
| 2 | Forest fires |  |  |  |  |
| 3 | Fire (community) |  |  |  |  |
| 4 | Flood |  |  |  |  |
| 5 | Windstorm |  |  |  |  |
| 6 | Thunderstorm |  |  |  |  |
| 7 | Hailstorm |  |  |  |  |
| 8 | Heavy rain |  |  |  |  |
| 9 | Sporadic rain |  |  |  |  |
| 10 | Landslide |  |  |  |  |
| 11 | Avalanche |  |  |  |  |
| 12 | Heat waves |  |  |  |  |
| 13 | Cold snaps |  |  |  |  |
| 14 | Diseases/Insects |  |  |  |  |
| 15 | Others |  |  |  |  |

**Module 6.2: Impact of Climate induced Natural Disasters or Events on Household Daily Life**

| S.N. | Type of natural  disaster or events  (please only ask  question based on  identified disaster  in 6.4) | Had you or your family member(s) been affected due to damage in physical infrastructure (road, irrigation schemes, etc.) due to the following disaster(s) over the past 15 years? | How many days on  average were you or your household members not able to work/ **unemployed** or experienced **food shortage** due to the following disasters in past 15 years? (days) | | In the past 15 years did you or any  of your family  member(s) bear any property loss? | If yes, please, write total property of loss in Rupees | Did any family member(s)  die due to the  impact of  following  disaster(s) over the past 15 years? | Death of family members over the past 15 | | | | |
| --- | --- | --- | --- | --- | --- | --- | --- | --- | --- | --- | --- | --- |
|  |  |  |  |  |  |  |  | years due to the following disaster(s) | | | | |
|  |  |  |  |  |  |  |  | Deaths by | | | Deaths | |
|  |  |  |  |  |  |  |  | age group | | | per sex | |
|  |  |  |  |  |  |  |  | 0-18  years | 19-59 years | 60  years or above | Male | Female |
|  |  | Affected..1, Seen but not affected ..2, No disaster..3 |  |  | Yes....1  No......2  (go to  G06) |  | Yes…1  No…2 |  |  |  |  |  |
|  |  |  |  |  |  |  |  |  |  |  |  |  |
|  |  |  |  |  |  |  |  |  |  |  |  |  |
|  |  | **G01** | **(UNE) G02** | **(FS) G03** | **G04** | **G05** | **G06** | **G07** | **G8** | **G9** | **G10** | **G11** |
|  |  |  |  |  |  |  |  |  |  |  |  |  |
| 1 | Drought |  |  |  |  |  |  |  |  |  |  |  |
| 2 | Forest fires |  |  |  |  |  |  |  |  |  |  |  |
| 3 | Fire (community) |  |  |  |  |  |  |  |  |  |  |  |
| 4 | Flood |  |  |  |  |  |  |  |  |  |  |  |
| 5 | Windstorm |  |  |  |  |  |  |  |  |  |  |  |
| 6 | Thunderstorm |  |  |  |  |  |  |  |  |  |  |  |
| 7 | Hailstorm |  |  |  |  |  |  |  |  |  |  |  |
| 8 | Heavy rain |  |  |  |  |  |  |  |  |  |  |  |
| 9 | Sporadic rain |  |  |  |  |  |  |  |  |  |  |  |
| 10 | Landslide |  |  |  |  |  |  |  |  |  |  |  |
| 11 | Avalanche |  |  |  |  |  |  |  |  |  |  |  |
| 12 | Heat waves |  |  |  |  |  |  |  |  |  |  |  |
| 13 | Cold snaps |  |  |  |  |  |  |  |  |  |  |  |
| 14 | Diseases/Insects |  |  |  |  |  |  |  |  |  |  |  |
| 15 | Others |  |  |  |  |  |  |  |  |  |  |  |

**Module 7: Disease and Health Impacts**

| **H01**. Has the incidence of illness increased in your family over the past 5 years? | Yes …… 1, No …… 2 (If no go to H03) | | | | | | Code: | | | | | |
| --- | --- | --- | --- | --- | --- | --- | --- | --- | --- | --- | --- | --- |
|  |  |  |  |  |  |  |  |  |  |  |  |  |
|  |  |  |  | |  |  |  | |  | |  | |
| **H02**. If yes, please provide the name of diseases experienced? | 1. | | ………………… | |  | | 2. | | | ………………………………… | | |
|  |  |  |  | |  | |  | |  | |  | |
|  |  | 3. | …………………… | |  | | 4. | | | ………………………………… | | |
|  |  |  | | |  | |  | |  | |  |  |
| **H03**. Have you noticed the incidence of any new diseases over | Yes=1, No=2 (if no go to H05) | | | |  | |  | | Code: | |  |  |
| the past 5 years? |  | |  |  |  | |  | |  | |  |  |
|  |  |  |  | |  | |  | |  | |  | |
| **H04**. If yes, please provide name of new diseases experienced in the | 1. | | ……………, . | |  | | 2. | | | ………………………………… | | |
| Family. |  | |  |  |  | |  | |  | |  |  |
|  | 3. | | ……………… | |  | | 4. | | | ………………………………… | | |
|  |  |  |  |  |  |  |  |  |  |  |  |  |
| **H05.** Has the incidence of airborne diseases increased in your family over the past 5 years? | Yes=1, No=2 | | | | | | Code: | | | | | |
| **H06:** If yes, please provide the name of diseases. | 1. 2. | | | | | | 3 4. | | | | | |
| **H05**. Has the incidence of vector-borne diseases increased in your family over the past 5 years? | Yes=1 No=2 (if no go to H07) | | | | | | Code: | | | | | |
|  |  |  |  |  |  |  |  |  |  |  |  |  |
|  |  |  |  | |  | |  | |  | |  | |
| **H06**. If yes, please provide the name of newly experienced diseases. | 1. | | ………………… | |  | | 2. | | | ………………………………… | | |
|  |  |  |  | |  | |  | |  | |  | |
|  |  | 3. | …………… | |  | | 4. | | | ………………………………… | | |
|  |  |  |  |  |  | |  | |  | |  |  |
| **H07**. Has the incidence of waterborne diseases increased in your family over the past 5 years? |  | Yes=1, No=2 (if no go to H09) | | | | | Code: | | | | | |
|  |  |  |  |  |  |  |  |  |  |  |  |  |
|  |  |  |  |  |  | |  | |  | |  |  |
| **H08**. Which waterborne diseases are most prevalent in humans compared to 5 years ago? (write the disease codes from below) * | 1 .……. | | | | | | | | 2. ……………………. | | | |
| **H09**. Has the incidence of food-borne diseases increased in your family over last 5 years? |  | Yes …1, No … 2 | | | | | | Code: | | | | |
|  |  |  |  |  |  |  |  |  |  |  |  |  |
|  |  |  |  |  |  |  |  |  |  |  |  |  |
| **H10**. Which food-borne diseases have been the most prevalent in your family over the past 5 years? |  | 1………………….. | | | | |  | |  | | 2. | ………………………….. |
|  |  |  |  |  |  |  |  |  |  |  |  |  |
|  |  |  |  |  |  |  |  |  |  |  |  |  |
|  |  |  |  |  |  |  |  |  |  |  |  |  |
| *Disease Codes: Diarrhea – 01, Dysentery – 02, Malaria – 03, Skin Disease – 04, Cold/Cough – 05, Fever – 06, Typhoid – 07, Asthma – 08, Jaundice – 09, Malnutrition related-10, Dengue – 11, Mental Disorder – 12, Chicken Pox – 13, Cholera – 14, Respiratory Disease=15, Viral encephalitis=16, Kalazar= 17, Water-borne & food-borne disease= 18, Measles=19, Mumps- 20, Rubella-21, Tuberculosis-22, Salmonella=23, Leptospirosis and Hantavirus=24 (Rodent-borne diseases), Others (specify) – 23, Write name of disease in the box | | | | | | | | | | | | |
|  |  |  |  |  |  |  |  |  |  |  |  |  |

**Module 8: Health Inaction Cost (Dropped)**

**Module 9: Impact of Climate Change in Air Quality and Water Resources (over the past 15 years)**

| **Description** |  |  | **Options** | | **Code** |
| --- | --- | --- | --- | --- | --- |
| **Jo1**. What changes have you observed in air quality at your locality? | Increased-1 | Decreased-2 | No changes-3 Do not know-4 | |  |
| **J02**. Have you felt any flow of hot air in your locality? | Yes-1 | No-2 |  | |  |
|  |  | | | |  |
| **J03**. What changes have you observed in the amount of water in streams, rivers, rivulets and springs? | Increased-1 Decreased-2 No changes-3 Do not know-4 | | | |  |
| **J04**. Have you noticed water quality deterioration in streams, rivers, rivulets  and springs? | Yes-1 No-2 No changes-3 Do not know-4 | | | |  |
|  |  |  |  |  |
|  | | | |  |
| **J05**. What changes have you observed in water levels in wells and ponds? | Increased-1 Decreased-2 No changes-3 Do not know-4 | | | |  |
|  |  | | |  |
| **J06**. Have you noticed water quality deterioration in wells and ponds? | Yes-1 | No-2 Do not know-3 | | |  |
|  |  |  |  |  |  |
| **J07**. Have any streams, rivers, rivulets, or springs dried up? | Yes-1 | No-2 | Do not know-3 |  |  |
|  |  |  |  |  |  |
| **J08**. Are any tube wells, wells or hand pumps drying up? | Yes-1 | No-2 | Do not know. 3 |  |  |
|  |  |  |  |  |  |
| **J09**. Have any of the wells, tube wells, or ponds dried up? | Yes-1 | No-2 | Do not know-3 |  |  |
|  |  | | | |  |
| **J10**.What changes have you observed in the amount of water in spouts? | Increased-1 Decreased-2 No change-3 Do not know-4 | | | |  |
|  |  |  |  |  |
| **J11**. Have any spouts dried up? | Yes-1 | No-2 | Do not know-3 |  |  |
|  |  | | | |  |
| **J12**. Has there been any change in the duration of water flow in piped water that you have been using on a daily basis? | Increased-1 Decreased-2 No change-3 Do not know-4 | | | |  |
|  |  |  |  |  |
|  |  |  |  |  |

**Module 10: Impact of Climate Change on Biodiversity over the Past 15 Years**

| **S.N** | **Description** | **Changes in species present**  *Have changed=1, Not changed=2, Do not know=3* | **Name of declining species (max. three)** | **Name of increasing species** | **Name of species no longer seen** | **Name of new species** |
| --- | --- | --- | --- | --- | --- | --- |
|  |  | **K01** | **K02** | **K03** | **K04** | **K05** |
| **1** | Trees/shrubs |  | 1. ………… 2. ……… |  | 1. …………. | 1.…………….... |
| **2** | Grasses |  | 1. ………… 2. ………….... | …………………… | 2.  …………………. | 2. ……………………. |
| **3** | Aquatic animals |  | 1. …………… 2. ………………. | ………………… | 3.  …………………. | 3. ……………………. |
| **4.** | Medicinal and non-timber forest |  | …………………… | ………………….. | …………………… | ……………………… |

**Module 11: Household Behavioral Factors**

| **Description** | **Options** | **Code** |
| --- | --- | --- |
| **L01**. What do you use to wash your hands before meals and preparing food? | *Mud-1, Soap-2, Water only-3, Ash-4, Do not wash-5* |  |
| **L02**. Do you wash all cooking utensils before and after cooking? | *Yes-1, No-2* |  |
| **L03**. Do you prefer lightly cooked food or deep cooked food? | *Lightly cooked-1, Deep cooked-2* |  |
| **L04**. What do you do with stale food? | *Eat again-1, Give to animals-2, Dispose on field-3* |  |
| **L05**. Where do you collect solid waste? | *Inside kitchen-1, Outside kitchen-2* |  |
| **L06**. What do you use to collect waste? | *Plastic bag-1, Open bin-2, Covered bin-3, Throw outside home-4* |  |
| **L07**. If household disposed of or stored outside the kitchen, can houseflies enter kitchen from waste? | *Yes-1, No-2* |  |
| **L08.** Do you drink properly treated clean water? | *Yes-1, No-2* |  |
| **L09**. Do you prefer boiled water or non-boiled water? | *Boiled-1, Non-boiled-2* |  |

**Module 13: (Dropped)**

**Module 14: Household Sociopolitical Access**

**P01**. Membership in political/social organizations:

1) Yes 2) No **(If no, go to P04)**

**P02**. If yes, how are you involved?

1) To build new organizations in your community 2) To gather people for demonstrations

3) Both (1) and (2) 4) Passive member

**P03**. If yes, how much time do you give to political/social organizations?

1) More than other community members 2) Based on my choice or using my leisure time

3) Don’t know 4) None

**P04**. Do you think that political parties or social organizations involve your community members more than other communities?

1) Yes 2) No

**P05** If yes, how do you measure the intensity of utilization of people from this community?

1) Very high 2) High 3) Moderate 4) Low 5) Very low 6) None

**P06**. Do political parties or social organizations use community people in their activities:

1) With payment 2) Without payment

**P07**. Do you feel any effects from the involvement of community people by political parties or social organizations?

1) Yes 2) No

**P08**. If yes, how do you rate the intensity of this effect?

1) Very high 2) High 3) Moderate 4) Low 5) Very low 6) None

**Thank you for your time and information.**
